# Celastrol Alleviates Chronic Obstructive Pulmonary Disease by Inhibiting Cellular Inflammation Induced by Cigarette Smoke via the Ednrb/Kng1 Signaling Pathway

**DOI:** 10.3389/fphar.2018.01276

**Published:** 2018-11-15

**Authors:** Ke Shi, Xi Chen, Bin Xie, Sha Sha Yang, Da Liu, Gan Dai, Qiong Chen

**Affiliations:** ^1^Department of Geriatrics, Xiangya Hospital of Central South University, Changsha, China; ^2^National Clinical Research Center for Geriatric Disorders, Xiangya Hospital, Central South University, Changsha, China; ^3^Respiratory Medicine, Xiangya Hospital of Central South University, Changsha, China; ^4^Department of Microbiology, Xiangya School of Medicine, Central South University, Changsha, China

**Keywords:** Ednrb, Kng1, chronic obstructive pulmonary disease, cigarette smoke exposure, inflammation, Celastrol

## Abstract

Chronic obstructive pulmonary disease (COPD) is a debilitating disease caused by chronic exposure to cigarette smoke (CS). Celastrol is a pentacyclic triterpenoid compound exhibits potent antioxidant and anti-inflammatory activities. Also it is presently known to protect against liver damage induced by type II diabetes. However, its role in COPD is unclear. In this study, we investigated the effects of Celastrol on cellular inflammation in mice exposed to CS and Beas-2B cells treated with CS extract (CSE). C57BL/6 mice and Beas-2B cells were randomly divided into three groups: control group, COPD or CSE group, and Celastrol treatment group. The COPD mice models were subjected to smoke exposure and cell models were treated with CSE. Bioinformatics analysis was performed to identify differentially expressed genes following treatment with Celastrol in COPD, the molecular networks was mapped by Cytoscape. The levels of inflammatory cytokinesinterleukin-8, tumor necrosis factor α, monocyte chemoattractant protein-1, and oxidative stress factors superoxide dismutase and catalase were measured by enzyme-linked immunosorbent assay. Hematoxylin and eosin staining to detect the injury of mouse lung tissue. mRNA and protein levels of Ednrb and Kng1 in the tissues and cells were measured by quantitative polymerase chain reaction (PCR) and western blotting, respectively. Apoptosis was measured by flow cytometry and TUNEL staining. Compared to mice in the COPD group, mice treated with Celastrol had significantly reduced levels of inflammatory cytokines interleukin-8, tumor necrosis factor α and monocyte chemoattractant protein-1 in the serum and bronchoalveolar lavage fluid, and significantly increased levels of oxidative stress factors superoxide dismutase and catalase. The same results were obtained at the cellular level using Beas-2B cells. Compared to the model groups, Celastrol reduced lung injury in mice and significantly reduced cellular apoptosis. Bioinformatics analysis showed that Ednrb is a target gene of Celastrol and differentially expressed in COPD. Quantitative PCR analysis showed that Ednrb expression in patients with COPD was significantly increased compared to that in healthy controls. Additionally, Celastrol effectively reduced Ednrb/Kng1 expression in both cell and animal models. Celastrol has a therapeutic effect on COPD and may alleviate COPD by inhibiting inflammation development by suppressing the Ednrb/Kng1 signaling pathway.

## Introduction

Chronic obstructive pulmonary disease, or COPD, is a destructive pulmonary disease characterized by incompletely reversible airflow obstruction. It is the third leading cause of death and fifth leading cause of chronic disability worldwide (Taivassalo and Hussain, 2016). The molecular mechanisms of COPD pathogenesis include alveolar epithelial or endothelial cell senescence, apoptosis, and chronic inflammation induced by oxidative stress (Fallica et al., 2014). While early-stage COPD can be prevented and treated, the disease can progress and deteriorate, eventually becoming incurable. Chinese herbal medicines with minimal adverse effects may provide new treatment strategies (Ito and Mercado, 2014), and some traditional Chinese medicines for treating COPD have been reported. Epigallocatechin gallate has been shown to inhibit chronic airway inflammation and abnormal airway mucus production by inhibiting the epidermal growth factor receptor signaling pathway (Liang et al., 2017). Silymarin has been shown to inhibit autophagy and the extracellular receptor kinase mitogen-activated protein kinase signaling pathway to reduce inflammatory responses (Li et al., 2016a).

Celastrol is a traditional Chinese medicine which was extracted from the roots of *Tripterygium wilfordii*, it is a pentacyclic triterpenoid compound that can effectively inhibit the nuclear factor-κB signaling pathway (Hu and Zhang, 2017; Chen et al., 2018). Its role and molecular mechanisms in cancer therapy have been extensively reported (Freudlsperger et al., 2013; Ouarssani et al., 2013). For example, in osteosarcoma, Celastrol achieves therapeutic effects by inducing the apoptosis and autophagy of cancer cells through the reactive oxygen species/c-Jun N-terminal kinase signaling pathway (Li et al., 2015). Additionally, Celastrol displays considerable anti-inflammatory activity (De Seabra Rodrigues Dias et al., 2017; Zhang et al., 2018) and can inhibit cellular inflammation by suppressing NLRP3 activity (Yu et al., 2017). Celastrol can also inhibit inflammation by inhibiting the expression of interleukin (IL)-1β (Li et al., 2016b). Although the anti-inflammatory effects of Celastrol have been studied in many diseases, there are no reports of its anti-inflammatory effects or its molecular mechanisms in COPD.

In this study, a COPD mouse model and cigarette smoke exposure (CSE)-induced cell model were used to examine the biological role of Celastrol in inflammation induced by smoke exposure and CSE, respectively. We also investigated whether reductions in inflammatory responses by Celastrol were achieved through inhibition of the Ednrb/Kng1 signaling pathway.

## Materials and methods

### Reagents

Celastrol (purity ≥98%) was purchased from Sigma (St. Louis, MO, USA) and dissolved in 10% dimethyl sulfoxide (Sigma). It was diluted with cell culture solution prior to treatment to ensure that dimethyl sulfoxide had no effect on the cells. Primers for monocyte chemoattractant protein (MCP)-1, caspase1, pdk4, and foxo1 were synthesized by Invitrogen (Carlsbad, CA, USA). Primary antibodies were purchased from either Abcam (Cambridge, UK) or Cell Signaling Technology (Danvers, MA, USA). Enzyme-linked immunosorbent assay (ELISA) kits for MCP-1, IL-10, catalase (CAT), and superoxide dismutase (SOD) were purchased from Immunoway (Plano, TX, USA), R&D Systems (Minneapolis, MN, USA), and Abcam.

### Clinical sample collection

Patients diagnosed with COPD at the Xiangya hospital of Central South University were selected as the study subjects, and the control group included individuals who were healthy as confirmed by health checkups during the same period. All patients included in the study signed informed consent forms and the study was approved by the medical ethics committee of Xiangya Hospital, central South University. All patients with COPD and healthy controls did not significantly differ in age or gender.

### Differential gene expression analysis

Gene expression data were downloaded from Gene Expression Omnibus (GEO), the microarray database of NCBI. The GSE84156 and GSE52509 datasets from GEO were selected for subsequent analysis. R language limma package was used to preprocess the GEO data and for differential gene analysis. Significantly differentially expressed genes were screened under the criteria of adj.P.val < 0.05 and |Log2FC| >1, i.e., the genes affected by Celastrol and differentially expressed in COPD were identified. The STITCH database was used to construct integrated gene network maps using the Celastrol target genes differentially expressed in COPD and genes involved in autophagy, oxidative stress, and inflammatory responses. The STRING website was used to predict genes downstream of the genes of interest.

### Cell culture

The normal human bronchial epithelial cell line Beas-2B was purchased from ATCC (Manassas, VA, USA). Beas-2B cells were cultured in medium (Gibco, Grand Island, NY, USA) containing 10% fetal bovine serum (Gibco), 100 U/mL penicillin, and 100 μg/mL RPMI-1640, and were maintained at 37°C in a saturated humidity atmosphere containing 5% CO_2_. The cells were passaged for three generations. When cell confluence reached 80–90%, the cells were digested and treated with 6% CSE+DMSO or 6% CSE+Celastrol (final concentration 5 μM) for 24 h. Normal cells were treated with 10% DMSO was a control. The methods for CSE preparation have been described previously (Ding et al., 2015).

### Animal treatment

Twenty-four 4-week-old C57BL/6 mice (18–20 g) were purchased from Hunan Slake Jingda Experimental Animal Co. Ltd. (Shanghai, China). The animals were randomly divided into three groups: control group (*n* = 8), model group (*n* = 8, CS exposure), and treatment group (*n* = 8; CS exposure+Celastrol treatment). Mice in the control group were housed under normal conditions. Mice in the model group were exposed to a smoke device system prepared in the laboratory. Treatments were performed as described previously (Zeng et al., 2018): 10 cigarettes/time, 30 min/time, 5 days/week, for a total of 12 weeks. Mice in the treatment group were intragastrically administered Celastrol (15 mg/kg) 2 h before smoke exposure. All animals were housed at a temperature of 22 ± 1°C, humidity of 65–70%, and light-dark cycle of 12/12 h. All animals were sacrificed at week 13. Blood was collected prior to sacrifice and serum was stored at −80°C. The study was approved by the medical ethics committee of Xiangya Hospital, central South University.

### Bronchoalveolar lavage fluid (BALF) and lung tissue collection

Bronchoalveolar lavage was performed on the left lung after the right lung was ligated. Repeated lavage was performed with 0.4 mL phosphate-buffered saline and fluid was collected a total of 3 times. The recovery rate was above 80%. The lavage fluid was collected in 1.5-mL tubes (placed on ice). The BALF solution was well-mixed and centrifuged (4,000 × g, 10 min, 4°C). The supernatant was aspirated into 1.5-mL tubes and stored at −80°C. The right lobe was dissected, stored in a cryogenic tube, and immediately placed in a liquid nitrogen tank. The left lobe was fixed with 4% paraformaldehyde and stored at 4°C.

### Hematoxylin and eosin (HE) staining

The right lung tissue was fixed with 4% paraformaldehyde, dehydrated, embedded in paraffin, cut into 4-μm sections, and used for HE staining. The results were observed under an inverted microscope (Olympus, Tokyo, Japan). Lung injury was scord according to the following criteria (Schingnitz et al., 2010): (1) alveolar congestion, (2) hemorrhage, (3) infiltration or aggregation of neutrophils in airspace or vessel wall and (4) thickness of the alveolar wall/hyaline membrane formation. For each subject, a 5-point scale was applied: 0, minimal (little) damage; 1+, mild damage; 2+, moderate damage; 3+, severe damage; and 4+, maximal damage. Points were added up and are expressed as median ± range of injury score.

### Apoptosis detection: tunel and flow cytometry assay

Tunel assay was performed using an apoptosis detection kit (Sigma) according to the manufacturer's protocol. 3,3′-Diaminobenzidine was used as the chromogenic substrate. Stained cells with brown nuclei were considered positive for apoptosis (Fujimoto et al., 2018). Five visual fields were randomly selected from each tissue section using a microscope, in which apoptotic cells were counted and the mean apoptosis rate was calculated. Apoptotic rate was calculated as the number of apoptotic cells divided by the number of total cells.

The apoptotic rate was measured by flow cytometry using the Annexin V-fluorescein isothiocyanate/propidium iodide kit (Solarbio, Beijing, China) according to the manufacturer's instructions. The samples were loaded onto a flow cytometer (BD Biosciences, Franklin Lakes, NJ, USA) for analysis (Qi et al., 2018).

### Detection of inflammatory cytokines by ELISA

The levels of cytokines (MCP-1, CAT, SOD, IL-8, TNF-a) in the serum, bronchoalveolar lavage fluid, or cell supernatant were measured with the ELISA kits and according to the manufacturer's protocol. Each sample was analyzed in triplicate wells. OD_450_ was calculated after background subtraction, and a standard curve was plotted. All samples were stored at −80°C before the experiment.

### Western blotting

Total cellular protein was extracted from the collected tissue or cell samples using RIPA lysate, and protein concentration was determined using a BCA protein assay kit (Thermo Fisher Scientific, Inc., Waltham, MA, USA). Total protein was separated by 10% sodium dodecyl sulfate-polyacrylamide gel electrophoresis and transferred to a polyvinylidene fluoride membrane. The membrane was incubated with 5% bovine serum albumin for 1 h at room temperature, after which primary antibodies (EDNRB, 1:1000; Kng1, 1:500, Abcam) were added, and the membrane was incubated overnight at 4°C. Secondary antibodies were added and the membrane was incubated at room temperature for 2 h. The developed films were scanned as grayscale images and analyzed using Image-pro plus 6.0 software (Media Cybernetics, Silver Spring, MD, USA) with GAPDH (GAPDH, 1:1000, Cell Signaling Technology) as the internal control. The expression levels of target proteins were evaluated as the ratios of the detected gray values of the target proteins to that of GAPDH.

### Real-time PCR

Trizol reagent (Invitrogen) was used to extract total RNA from the tissues or cells. First-strand cDNA was synthesized using a PrimeScript™ II 1st Strand cDNA Synthesis Kit (Takara, Shiga, Japan). Real-time PCR was performed according to the protocol of the SYBR Green PCR Mix (Takara) using the 7900HT Fast Real-Time System (Applied Biosystems, Forster City, CA, USA). The sequences of the primers were: for Ednrb(GenBank: L06623.1): sence, 5′-GGGCTGCAGGTTTCGACC-3′ and antisence, 5′-CTGCAAACGCTAATACCGCC-3′; for Kng1(GenBank: BC060039.1): sence, 5′-CCTTTGGAATGGTGATACCG-3′ and antisence, 5′-CGCAAATCTTGGTAGGTGGT-3′; for caspase1(GenBank: NM_001257118.2): sence, 5′-TTTCCGCAAGGTTCGATTTTCA-3′ and antisence, 5′-GGCATCTGCGCTCTACCATC-3′; for GAPDH(GenBank: NM_002046.6): sence, 5′- CGGAGTCAACGGATTTGGTCGTAT-3′ and antisence, 5′-AGCCTTCTCCATGGTGGTGAAGAC-3′. The PCR conditions were as follows: 95°C for 3 min; 95°C for 10 s, 60°C for 30 s, for a total of 40 cycles. GAPDH was used as internal control and relative gene expression was calculated using 2^−ΔΔ*Ct*^ (Livak and Schmittgen, 2001).

### Statistical analysis

All values are expressed as the mean ± SEM (*n* = 3). Differences between the experimental group and control group were analyzed by Student's *t*-test. Analysis of variance was used for triplicate measurements. A *p* < 0.05 was considered to indicate statistical significance. Statistical analysis was performed using SPSS software 19.0 (SPSS, Inc, Chicago, IL, USA).

## Results

### Celastrol reduced lung inflammation and cellular apoptosis in mice with COPD

**Figure 1:**
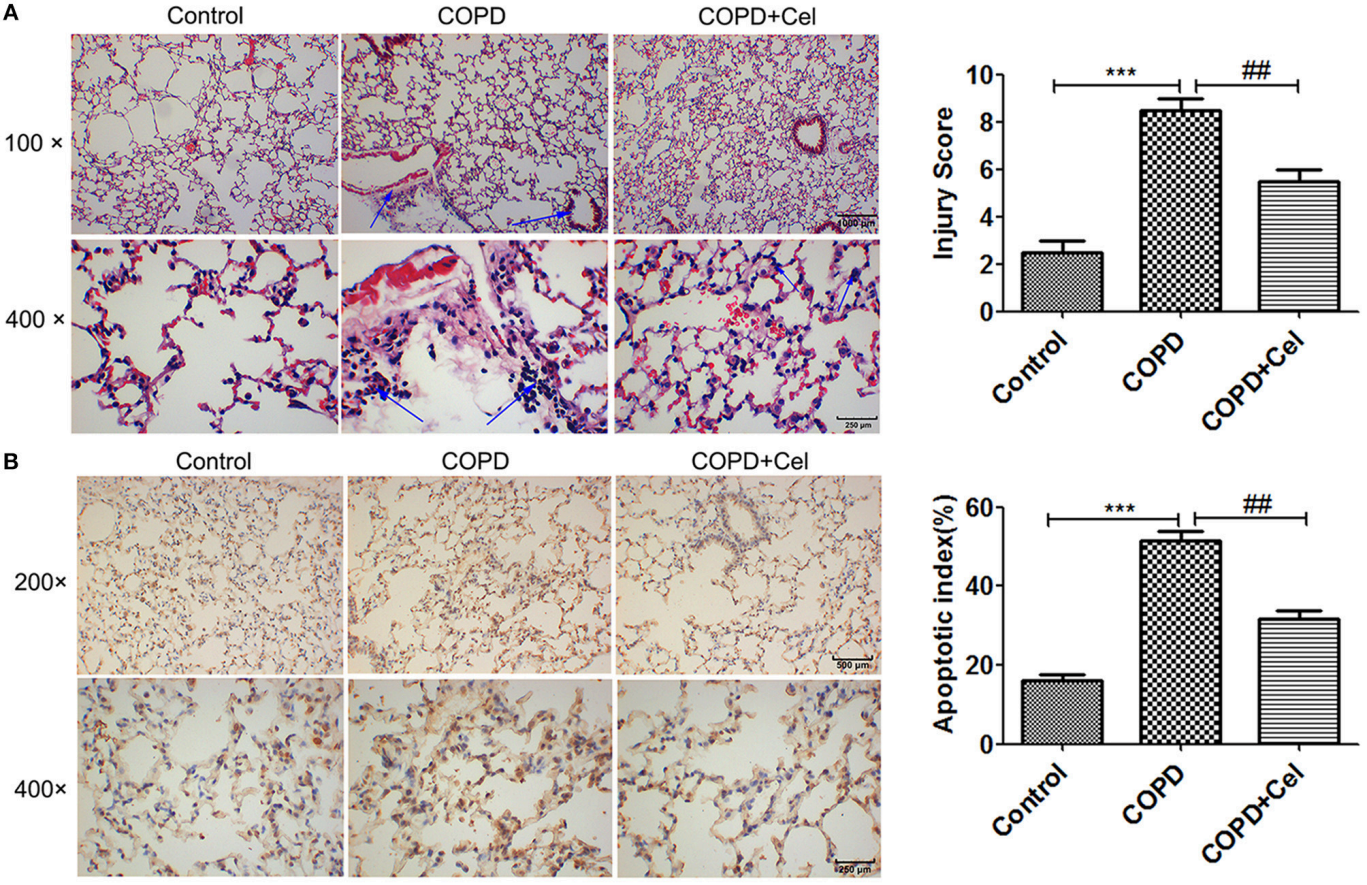
Pathological examination of mouse lung tissue. **(A)** HE staining of mouse lung tissue and viewed under the light microscope (× 100,scale bars = 1000 μm or × 400,scale bars = 250 μm). **(B)** Apoptosis in mouse lung tissue detected by TUNEL assay and histogram of the apoptosis rate of cells in mice lung tissue amongst control, COPD and COPD+celastrol groups (× 200, scale bars = 500 μm or × 400, scale bars = 250 μm. Blue arrow indicates the site of inflammation in **(A)**; the condensed and fragmented nuclei are indicated with arrows in **(B)**. Values are expressed as the mean ± SEM (*n* = 5); ^*^p < 0.05, ^**^p < 0.01, ^***^p < 0.001 with respect to the control group; ^#^*p* < 0.05, ^##^*p* < 0.01 with respect to the COPD group.

To investigate whether Celastrol has a protective effect against COPD, we constructed a COPD mouse model and damage to the lung tissue was observed by HE staining and TUNEL assay. The results of HE staining showed that smoke exposure aggravated inflammation in the lung tissues of mice, whereas treatment with Celastrol reduced tissue damage and inflammation; also the COPD group exhibited lung parenchymal destruction and airspace enlargement in the COPD group as compared with the control group, celastrol treatment group changed the symptom (Figure 1A). Additionally, TUNEL staining demonstrated that the apoptotic rate of cells in the COPD group were significantly higher than the control group (*p* < 0.001), but the apoptotic rate of cells in the COPD+cel group was markedly lower compared with COPD group (*p* < 0.01) (Figure 1B).

### Celastrol altered the levels of cellular inflammatory factors

**Figure 2:**
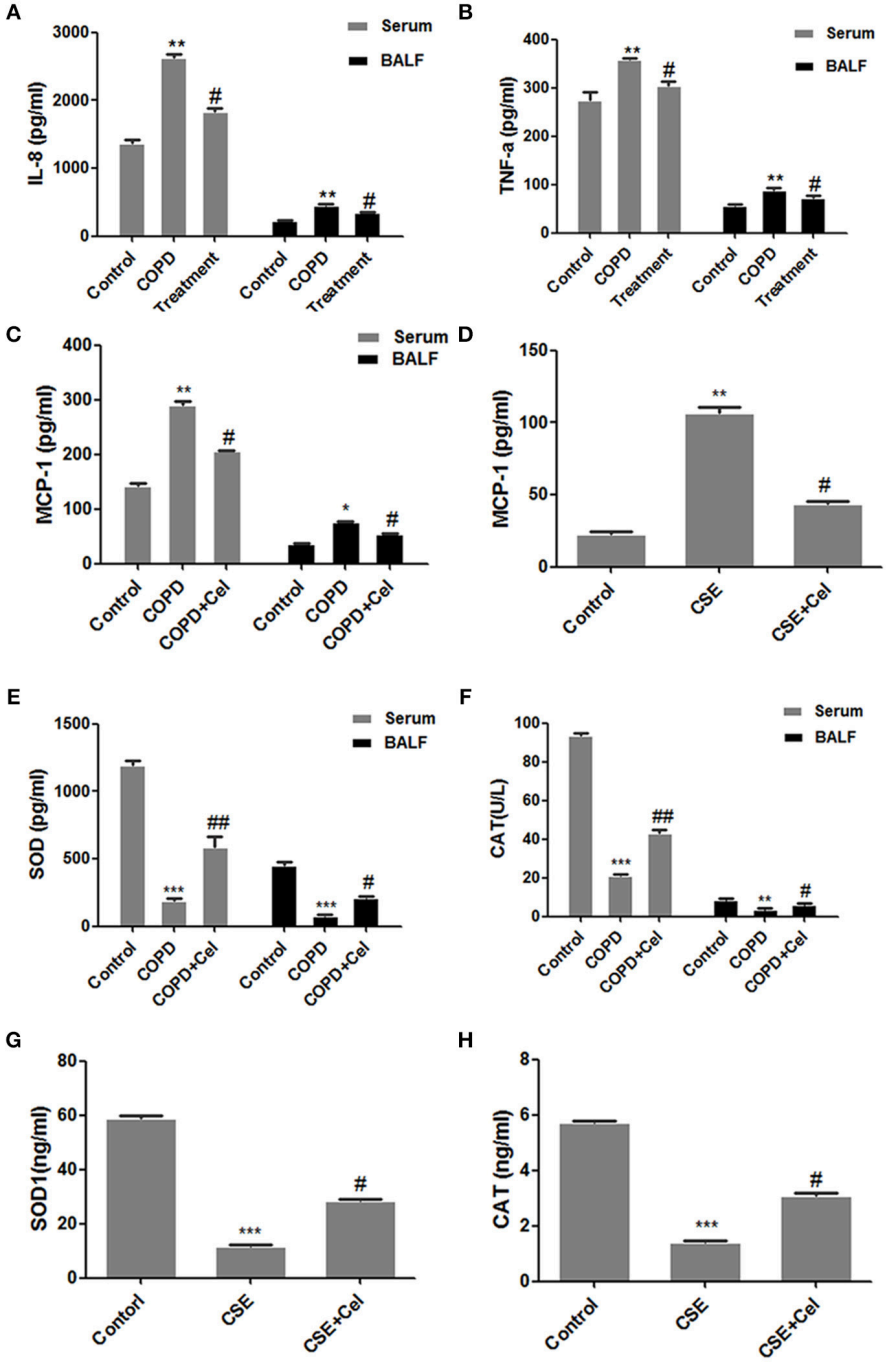
Detection of inflammation- and oxidative stress-related factors. **(A)** IL-8 levels in mouse serum and bronchoalveolar lavage fluid (BALF). **(B)** TNF-a levels in mouse serum and bronchoalveolar lavage fluid (BALF). **(C)** MCP-1 levels in mouse serum and BALF. **(D)** MCP-1 levels in the supernatant of Beas-2B cells. **(E)** SOD levels in mouse serum and BALF. **(F)** CAT levels in mouse serum and BALF. **(G)** SOD levels in the supernatant of Beas-2B cells. **(H)** CAT levels in the supernatant of Beas-2B cells. Each result was derived from three independent experiments. Control group were treated with DMSO; CSE group were treated with CSE and DMSO; CSE + celastrol were treated with CSE and celastrol. Values are expressed as the mean ± SEM (*n* = 3); ^*^p < 0.05, ^**^p < 0.01, ^***^p < 0.001 with respect to the control group; ^#^*p* < 0.05, ^##^*p* < 0.01 with respect to the CSE group or COPD group.

Next, we established a cell model of COPD and cell was treated with Celastrol. The level of inflammatory cytokines IL-8, TNF-a, and MCP-1 and oxidative stress factors SOD and CAT in the mouse serum and BALF as well as the supernatant of human bronchial epithelial cells were detected by ELISA (Figure 2). Compared to that in the control group mice, IL-8, TNF-a, and MCP-1 in the serum and BALF in COPD group mice was significantly increased (*p* < 0.05). Celastrol reduced the level of the cytokines IL-8 and MCP-1 induced by CS (*p* < 0.05) (Figures 2A–C). SimilarlyMCP-1 expression in the supernatant of cells in the CSE group was significantly higher than that in the control group (*p* < 0.05). In the Celastrol treatment group, MCP-1 expression was lower than that in the CSE group (*p* < 0.05) (Figure 2D). Additionally, the expression of oxidative stress factors SOD and CAT showed an opposite trend as the inflammatory factors. SOD and CAT levels in the serum and BALF were significantly lower in COPD group mice than in the control group mice, whereas Celastrol treatment reversed the decrease in SOD and CAT levels (Figures 2E,F). The same results were obtained at the cellular level in Beas-2B cells (Figures 2G,H).

### Celastrol reduced apoptosis

**Figure 3:**
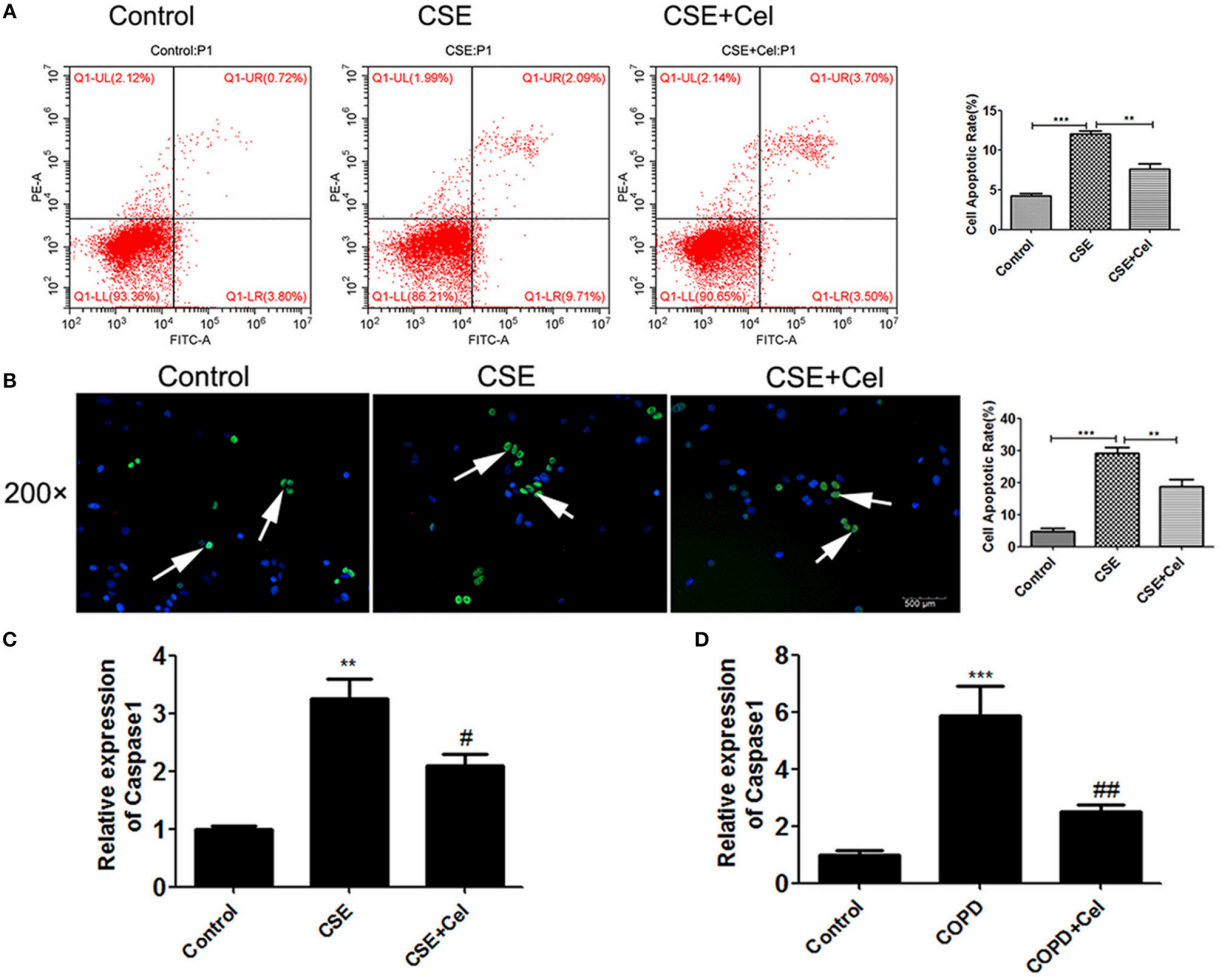
Effects of Celastrol on apoptosis. **(A)** Detection of apoptosis by flow cytometry. **(B)** Detection of apoptosis by TUNEL assay (× 200, scale bars = 500 μm). **(C)** mRNA expression of the apoptosis-related factor caspase-1 in cells. **(D)** mRNA expression of caspase-1 in mouse lung tissue. Each result was derived from three independent experiments. The condensed and fragmented nuclei are indicated with arrows. Values are expressed as the mean ± SEM (*n* = 3) ^**^p < 0.01, *p* < 0.001 with respect to the control group; ^#^*p* < 0.05, ^##^*p* < 0.01 with respect to the CSE group or COPD group.

Previous studies have shown that Celastrol can reduce apoptosis in the lung tissues of mice with COPD. To verify that Celastrol can exert the same effect at the cellular level, we performed flow cytometry and TUNEL assays to detect apoptosis of human bronchial epithelial cells treated with CSE and Celastrol. We found (Figures 3A,B) that CSE significantly promoted apoptosis (*p* < 0.05), while Celastrol effectively inhibited apoptosis. q-PCR was performed to detect expression of the apoptosis-related factor caspase-1 in human bronchial epithelial cells and mouse lung tissue. The results showed that caspase-1 expression was significantly higher in the CSE group than in the control group and significantly lower in the Celastrol treatment group than in the model group (Figures 3C,D).

### Ednrb was highly expressed in patients with COPD

**Figure 4:**
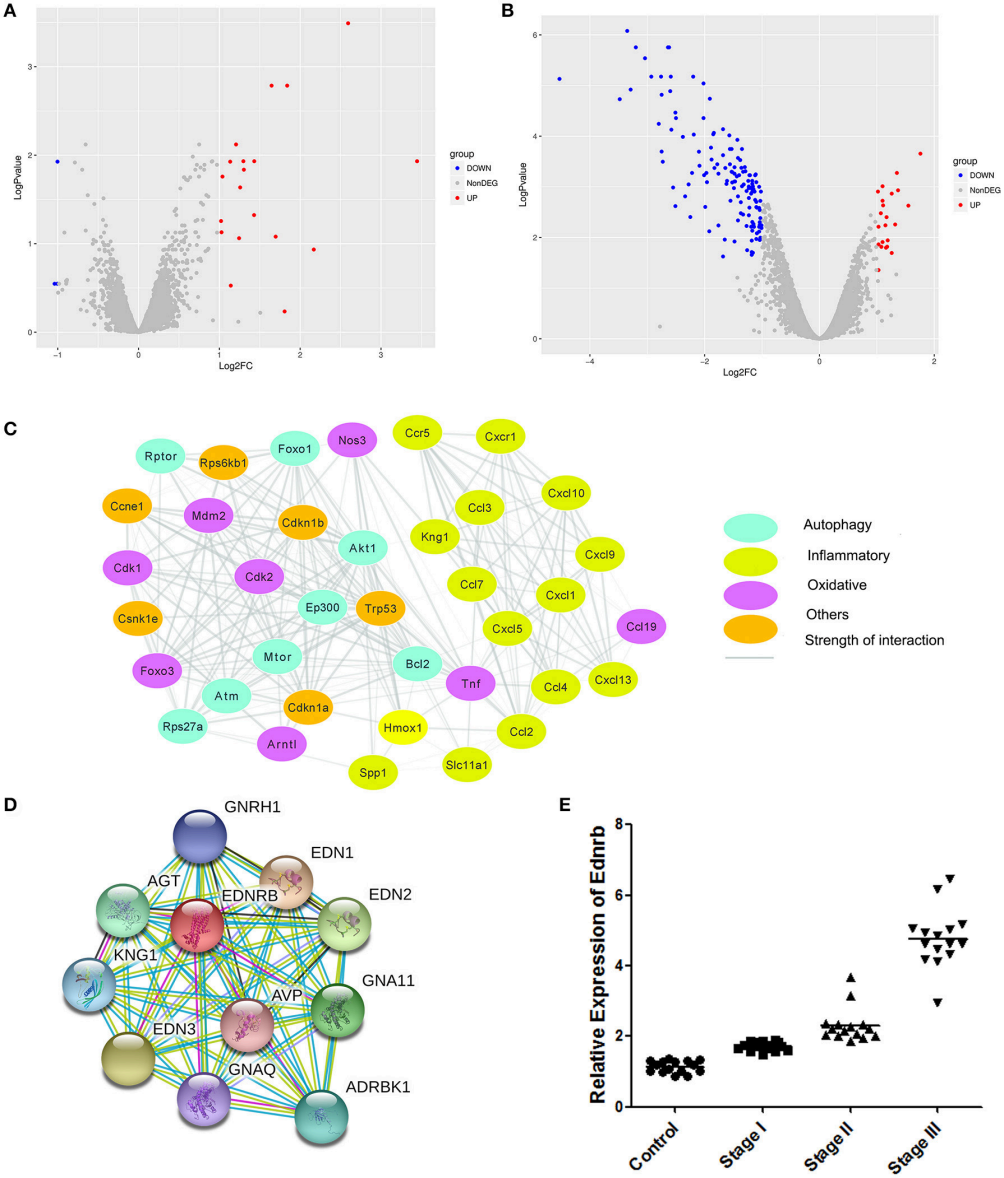
Identification of differentially expressed genes. **(A)** Differentially expressed genes between mice treated with Celastrol and normal mice. **(B)** Differentially expressed genes between mice with COPD and normal mice. **(C)** Gene network map of differentially expressed genes and genes involved in autophagy, oxidative stress, and inflammatory responses. **(D)** Gene network map of Ednrb and related genes. **(E)** Expression of Ednrb in clinical samples.

To investigate the molecular mechanism of the effects of Celastrol on COPD, we analyzed differences in gene expression in mice treated with Celastrol and control group mice (GSE84156). A total of 29 differentially expressed genes (abs(Log2FC) > 1, Adjusted_*p* value < 0.05) were identified (Figure 4A). Differences in gene expression in mice with COPD and normal mice were also analyzed (GSE52509), and 134 differentially expressed genes (abs(Log2FC)>1, Adjusted_p value < 0.05) were identified (Figure 4B). Comparison of the datasets revealed 58 Celastrol target genes that were also differentially expressed in COPD. Among these genes, 11 were associated with autophagy, oxidative stress, and inflammation Table 1. Using the STICH database, we found that these differentially expressed genes formed an integrated network with the genes involved in autophagy, oxidative stress, and inflammatory responses (Figure 4C). Additionally, among the genes in the nodes, the differentially expressed inflammation-related gene Ednrb was found to be upstream of another inflammation-related gene, Kng1 (Figure 4D). This indicates that Celastrol alleviates COPD by regulating cellular inflammation through Ednrb/Kng1. Therefore, we performed q-PCR to detect the expression of Ednrb in patients with COPD. The results showed that as the severity of COPD increased, the expression of Ednrb also significantly increased (*p* < 0.05, Figure 4E).

**Table 1:** Genes differentially expressed after Celastrol treatment and associated with autophagy, inflammation, and oxidative stress in COPD.

**Gene name**	**Type**
Pdk4	Autophagy
Cd14	Inflammatory
Ednrb	Inflammatory
Pglyrp1	Inflammatory
Tek	Inflammatory
Tlr7	Inflammatory
Cyplb1	Oxidative
Nqo1	Oxidative
Rhob	Oxidative
Sesn1	Oxidative
Slc7a11	Oxidative

### Celastrol inhibited Ednrb/Kng1 expression and reduced cellular inflammation

**Figure 5:**
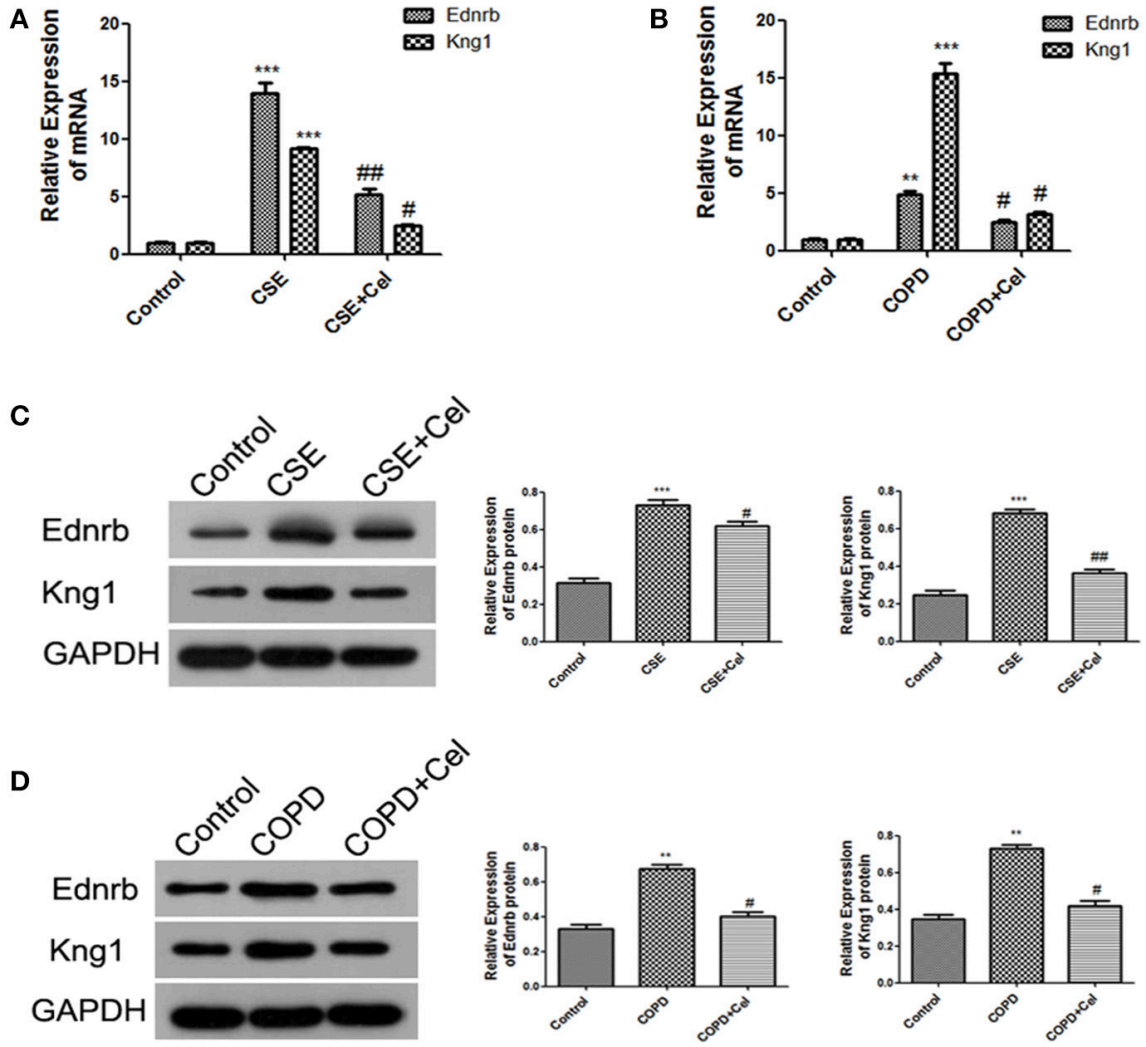
Celastrol inhibited the Ednrb/Kng1 signaling pathway. **(A)** Detection of mRNA expression levels of Ednrb and Kng1 in Beas-2B cells by q-PCR. **(B)** Detection of mRNA expression levels of Ednrb and Kng1 in mouse lung tissue by q-PCR. **(C)** Detection of protein expression levels of Ednrb and Kng1 in Beas-2B cells by western blotting. **(D)** Detection of protein expression levels of Ednrb and Kng1 in mouse lung tissue by western blotting. Each result was derived from three independent experiments. Values are expressed as the mean ± SEM (*n* = 3) ^**^p < 0.01, ^***^p < 0.001 with respect to the control group; ^#^*p* < 0.05, ^##^*p* < 0.01 with respect to the CSE group or COPD group.

Our results showed that Ednrb expression was significantly increased in patients with COPD (Figure 4E). Since our previous results (Figure 4D) predicted that Ednrb regulates the expression of Kng1, we examined whether Celastrol affected cellular inflammation by regulating Ednrb/Kng1. Therefore, we performed q-PCR and western blotting to detect the expression of Ednrb and Kng1 in the tissue and cell samples. The same results were observed at the cellular and animal levels (Figure 5), in which Celastrol reduced the high expression of Ednrb induced by CSE. Similar results were obtained for the expression of Kng1.

## Discussion

COPD is an inflammatory disease defined as the presence of incomplete reversible air flow obstruction which has become one of the global public health problems, it could afflict more than 600 million people worldwide (PMID: 23902484). Previous study shows that traditional Chinese medicine which have anti-inflammatory effects may be a potential drug for COPD treatment (PMID: 28932196\PMID:27874084).

Celastrol is a pentacyclic triterpenoid compound extracted from the roots of *T. wilfordii* and exerts anti-inflammatory effects in diseases through various molecular mechanisms have been demonstrated (Chen et al., 2018; Coulibaly et al., 2018). Although celastrol has a unfavorable physicochemical and pharmacokinetics properties such as low solubility, poor bioavailability, and systemic toxicity, are limiting its therapeutic application, previous study have explored many strategy to overcome these issues (PMID:29191042\PMID: 26586945\PMID: 28905613\PMID: 22982018). The role of Celastrol in the treatment of lung diseases has received widespread attention. Studies of lung cancer have shown that Celastrol promotes the sensitivity of lung cancer cells to cisplatin. Additionally, Celastrol can inhibit pulmonary fibrosis caused by bleomycin by regulating transforming growth factor-β/Smad (Divya et al., 2018). IL-8(Interleukin-8), TNF-a (tumor necrosis factor α), MCP-1(monocyte chemoattractant protein-1) which were increased after CS exposure (PMID: 23875733/PMID: 30258361). Oxidative stress response can aggravate airway inflammation, previous research showed that the SOD and CAT decreased in the patients with COPD (PMID: 25685791/PMID:23683270); also both the SOD and CAT the same in the animal models of COPD (PMID: 28108387\PMID: 28932196). Our study shows that severe lung injury occurred in the mouse model of COPD. Additionally, levels of the inflammatory cytokines IL-8, TNF-a and MCP-1 in the mouse serum and BALF were significantly increased, while levels of the oxidative stress markers SOD and CAT were significantly reduced. The results are consistent with those of previous reports (Tavilani et al., 2012; Liang et al., 2017), in which Celastrol inhibited changes in inflammatory factors and oxidative stress markers and showed anti-inflammatory and antioxidant effects. Celastrol also exerted the same effects in CSE-treated cell models. Apoptosis may affect cellular inflammation induced by CS (Long et al., 2018). Recent studies showed that the necrosis inhibitor NEC-1 can significantly reduce the incidence of neutrophilic airway inflammation in CS-exposed mice (Pouwels et al., 2016). Decreased mTOR gene expression has been shown to promote CS-induced inflammation and apoptosis (Wang et al., 2018). In our study, apoptosis was significantly higher in CS-exposed mice and CSE-treated Beas-2B cells than in the respective control groups. These results indicate that Celastrol inhibits apoptosis caused by CS exposure or CSE, as well as reduces inflammation, which are consistent with previous studies.

Bioinformatics analysis in the present study revealed low expression of the endothelin receptor type B (Ednrb) gene after Celastrol treatment and high expression in COPD. Ednrb, also known as endothelin type B receptor, is a G protein-coupled receptor primarily expressed on endothelial cells. It can stimulate vasodilation and the release of the anti-proliferative mediator prostacyclin (Zahedi et al., 2015; Chen et al., 2016). Expression of Ednrb in patients with COPD was significantly higher than that in healthy individuals, suggesting that Ednrb plays a role in the progression of COPD.

Kng1 encodes high-molecular weight kininogen proteins and plays an important role in inflammation, blood pressure regulation, and coagulation (Furniss et al., 2014; Wang et al., 2017). Studies showed that deletion of Kng1 protein in ischemic mice reduced thrombosis and inflammation (Langhauser et al., 2012). Also Kng1 as a pro-inflammatory cytokine, Which promotes the development of cellular inflammation (Langhauser et al., 2012; Kata et al., 2017). In this study, STRING database revealed that Kng1 is a downstream target gene of Ednrb. Additionally, Kng1 and Ednrb expression was increased significantly in COPD models created by CS exposure or CSE treatment, and Celastrol significantly lowered Ednrb and Kng1 expression in these models. But we should take more work to underly the mechanism of Ednrb/Kng1 on cellular inflammation and apoptosis on the further.

In summary, the *in vivo* and *in vitro* experiments in the present study showed that Celastrol alleviated COPD. It may play a role in reducing CS-induced inflammation of the lungs by inhibiting cellular inflammation and apoptosis induced by CSE via suppression of Ednrb/Kng1. However, the molecular mechanisms underlying the effect of Ednrb/Kng1 on cellular inflammation and apoptosis require further investigations.

## Author contributions

QC designed the work and offered funds. KS designed the work, performed the experiment and submitted the manuscript. XC and BX performed the experiment and collected the data. SY, DL, and GD analyzed data and modified the manuscript.

### Conflict of interest statement

The authors declare that the research was conducted in the absence of any commercial or financial relationships that could be construed as a potential conflict of interest.
